# Supplementary material for: Targeted analysis of dyslexia-associated regions on chromosomes 6, 12 and 15 in large multigenerational cohorts
Source: PLoS One. 2025 May 27;20(5):e0324006. doi: 10.1371/journal.pone.0324006 (PMC12112411; doi:10.1371/journal.pone.0324006)
Supplement: Attachment — Submitted filename: Response to Reviewers 3 May2024.docx [file pone.0324006.s007.docx]

May 3, 2024

Madelon van den Boer

Academic Editor
PLOS ONE

Dear Dr. van den Boer:

Thank you and the two reviewers for your thoughtful comments and suggestions. Below we itemize our responses.

You commented that the manuscript is “targeted to a very specific readership, with quite a lot of knowledge/experience about/with genetics research” and suggested we “consider small adjustments in order to make the paper more accessible to a broader audience, for example by stressing the added value of this approach in studying dyslexia, as well as some more information about the lines of evidence (e.g. familial aggregation, linkage and association studies, copy number scan, structural chromosome rearragements) and the effects of genes (e.g. ciliogenesis)”. PLoS One is unusual among journals for the enormous breath of its content. With respect to our manuscript, from our involvement in the community of scientists studying dyslexia and related disorders, we feel that people who do not have a deep knowledge of statistical and/or molecular genetics will still understand the overall approach, the advantage of more comprehensive sequencing, and the results. Adding even a brief description of each type of study - familial aggregation, linkage and association studies, copy number scan, and structural chromosome rearrangements – would lengthen the paper unnecessarily as we cite references to papers that use these approaches and describe them in detail. We do include a statement indicating the advantage/power of our sequencing approach over other types of investigations: *“This technology allows us, in this current multi-site study, to investigate the potential role of variants of smaller effect size, non-coding variants, and sample heterogeneity as possible explanations for previous variable results in ROIs implicated in dyslexia.”*

**Reviewer #1:** This is a well-written manuscript describing targeted analyses of previously reported dyslexia-associated genes and loci. Overall, the approach and methodologies are sound. The results, while not surprising, confirm the soundness of the approach, building on and expanding previous findings. The work is a significant contribution to the field which overall, lacks vigorous independent replication.

We thank the reviewer for these comments, which clearly articulate our major goal in carrying out the project!

There are some concerns that should be addressed:
1. On page 13, lines 262 – 268, section on Ancestry adjustment. More details should be included in the main text, such as the percentage of participants with self-reported ancestry and available SNP array genotype data for KING estimation. Of note, only 251 children in the SK data set and 532 individuals in the UW data set have existing SNP array genotype data to estimate ancestry - the SNP-based ancestry of nearly half of the participants in the study is unavailable. The authors should make note of the potential inaccuracy of self-reported ancestry. In addition, a simple European/non-European indicator may not be adequate to adjust for potential population stratification. Sensitivity analyses that include a more specific ancestry assignment as a covariate are suggested.

We have added the percentage of people with SNP-based ancestry in the results paragraph (p16). Self-reported ethnicity agrees well with SNP-based ancestry in this dataset, and we have added a line to that effect. We added two sentences on page 17 pointing out that the non-European populations are too small for a more stratified analysis and that sensitivity analyses done in the early stages of this work indicated that the simple Eur/non-Eur variable was appropriate.

2. On Page 13, lines 278-281, two different phenotype adjustments (age, sex, with/without VIQ) are described. On Page 13, lines 264-265, the authors also describe the use of a European/non-European indicator to adjust for ancestry. Please confirm the covariates adjusted in the analysis.

Thank you for pointing this out, we have clarified that there was an ancestry adjustment in both models (page 13).

3. On page 14 lines 284-285, the authors describe using linear regression to adjust for covariates. Did the authors check whether the original phenotypes followed the assumptions of linear regression?

Previous analyses in the UW data set have demonstrated that these models are appropriate to these phenotypes. We have added a sentence and citations to this effect (page 14).

4. Was there an adjustment for study site in the analysis?

Yes. Adjustment for study site was described in the original manuscript in the first sentence in “association testing” page 14. Phenotypes were adjusted separately within each data set, which effectively allows for a site-specific effect. We have added a sentence to make this point explicit for non-statistical readers.

*This effectively allows for site specific effects.*

5. Was there an adjustment for SES?

No. This (and other potential environmental covariates) was not available. We added a statement to this effect on page 14.

6. On page 14 lines 289-290, they mentioned that they used “data set-specific variances” in their analysis. What is this exactly? Was it used to account for difference between datasets.

The residual variance was allowed to differ between data sets. We have restated this (hopefully more clearly) on page 14.

7. On page 15 lines 311-314, the authors mentioned that they used a p-value of 0.0025 which was derived by 0.01 divided by 20. But 0.01/20=0.0005. This is confusing. What exactly was used as the cutoff for aggregate tests?

Thank you for pointing this out. This was a typographical error. Our target significance level was 0.05 and after Bonferroni correction for 20 tests, we have p<0.0025 as our threshold. We have corrected this on page 16 and in supplemental table 6.

8. The determination of threshold for the single-variant tests is unclear. The authors considered the number of candidates out of 25000 genes instead of the number of SNPs for testing. Since SNPs within the same gene are in high LD, the authors may need to consider the effective number of independent markers (Me) for the adjustment of multiple testing (Li, 2012).

From this comment, it appears that the reviewer may have been confused by our different corrections for multiple testing. We have two different corrections: one for the higher-frequency individual markers and one for the aggregate tests for the rare variants within the genes evaluated. To try to make this more understandable, we broke this topic out into its own paragraph at the end of the section and have attempted to clarify our multiple-test adjustments in both settings. In short: we do include LD block-effects for calibrating the significance of individual-variant tests through use of reference-sample thresholds, just as do GWAS in general. We do not include LD-block effects for the aggregate variant tests. Both situations take into account the number of genes/regions being independently tested.

For convenience we excerpt the edited text here:

*We determined significance thresholds for statistical testing as follows. A significance threshold for single-variant tests must account for the effects of LD blocks (e.g., Li et al 2012). Such thresholds do not change with increasing marker density (van den Berg 2019), but do depend on the population involved, due to differences in LD between populations (Li et al 2012). A study using 1KGP Phase 3 data showed that in European samples, a genomewide threshold of 9.26 × 10^-8^ is most appropriate for single-variant tests with a target type I error rate of 0.05. We used this genomewide threshold and scaled it to account for the approximate fraction of the genome under analysis. The present study involved 5 genes, compared to approximately 25000 in a full GWAS, so we use p < 4.63 × 10^-4^ (25000/5 × 9.26 × 10^-8^) as a stringent significance cutoff for single-variant tests. This allows for both the number of genes evaluated, and the presence of LD blocks in those gene regions. For aggregate testing, since the gene-region is the unit of analysis independent of presence/absence of LD blocks in the gene-region, further adjustment for the number of LD-blocks is not warranted. To achieve a type I error rate of 0.05, we therefore used a p-value of 0.0025 as the cutoff for aggregate tests, motivated by dividing 0.05 by 20 for a simple Bonferroni correction using the number of gene-region tests performed (4 tests for each of 5 genes). We did not adjust test thresholds for analysis of multiple traits since current studies do not typically do so.* *The limited literature to date shows no evidence for an increased false-positive rate, with the advantages of using multivariate and/or pleiotropic models falling primarily on the side of potential increase of power to detect true, but weak, associations (Julienne et al 2021).*

9. The thresholds for both the single-variant test and the Burden test didn’t adjust for the number of phenotypes. Given the strong correlations between the phenotypes, perhaps the effective number of phenotypes should be considered.

We did not adjust test thresholds for analysis of multiple correlated traits since current studies do not typically do so. The limited literature to date shows no evidence for an increased false-positive rate, with the advantages of using multivariate and/or pleiotropic models falling primarily on the side of potential increase of power to detect true, but weak, associations (Julienne et al 2021).

The last two sentences of the edited paragraph we excerpt above (page 16) include this information.

10. The authors conducted rare variant analysis by grouping SNPs based on their location relative to each candidate gene. We also suggest grouping SNPs within each gene based on functional annotations, such as loss-of-function and deleterious missense variants.

The following comment is for the reviewer; no edits were made to the text:

The vast majority of the variants we identified are in non-coding sequence (by design, we sequenced exons and regions of open chromatin). Those in non-coding sequence are not simple to annotate – combing available databases is time consuming and typically involves investigator examination of one SNP at a time. Thus, it is not easy to sort non-coding SNPs by annotation. Also, there were very few exonic variants in any of the genes considered, so binning based on SIFT or polyphen predictions would have resulted in sample sizes that are too small to be informative.

**Reviewer #2: # summary**
In this study, Chapman et. al. examine three dyslexia regions of interest in chromosomes 15 (DYX1), 6 (DYX2), 12 through targeted sequencing in a large sample of over 2,100 individuals from multiple families.
The article is well written and accurate. There are a few minor details that should be checked, as specified below.

\# minor comments
- short title: edit it to make it self contained, for instance replace MIP with targeted

Done, thank you.
- abstract, line 60: why does the data support an oligogenic model (vs a polygenic model)?

There is no sharp boundary between these two models. However, in the discipline of quantitative genetics that coined these terms, a polygenic model implies that genetic variation attributable to variation in individual genes (or other inherited units) is miniscule, and most genes contribute a tiny bit to the trait variance. In contrast, an oligogenic model implies multiple genes that may contribute through small, but non-trivial, contributions to the trait variance. Thus, what we find: - small number of sites that provide measurably different allelic effects – is more compatible with the oligogenic than the polygenic model.

- abstract, line 58: for consistency to how the rest of the results were reported, specify that the association between GRIN2B and spelling occurred with and without VIQ adjustment.

Thank you, we clarified this.

- introduction: page 5, line 93: when arguing for the locus in chromosome 12q, what does "most convincing" mean? this is better explained later on, both in the results and in the discussion, but I feel that it's necessary to mention the evidence supporting this locus as well when presenting the few selected genomic regions for this study.

We edited this sentence for clarity.

*A region on chromosome 12p provides evidence for linkage for a phonological non-word memory phenotype in the UW cohort (49), and harbors variants associated with dyslexia in other data sets (118-120).*

- methods:
- it is mentioned that the participants were selected for studies of dyslexia, and that there were both probands and related individuals, but it is not clear how many dyslexic individuals were included in the study, or what the distribution of the quantitative phenotypes looks like.

There is information in the supplement regarding proband qualification, which is as close as we can get to a binary dyslexia diagnosis. We have copied the information about proband definition into the main body of text, in both UW and SK cohorts. References are provided for previous analyses of both of these data sets. Information about proband definition was already there for the Houston cohort, and it is now highlighted.

- please specify how the genetic ancestry was defined for individuals with genotyping data was available. There is a reference in line 263 to "KING estimation", but it would be good to specifcy that this is referring to genetic ancestry estimation.

We added the word “ancestry” to this line (now 285) to clarify.

- I understand that some of the people that were sequenced did not have genotyping data (because genetic ancestry could not be defined for all the individuals), but
- page 8, lines 192-193: it is unclear whether parents were included in the present study (given the lack of phenotypic data on them?). If not, please state it clearly.

All individuals with both phenotype and genotype data were included in the analysis, as described in Table 1. To clarify we added a sentence to page 8.

*Children and related adults with both trait and genotype data were included in the analyses.*

- methods/(supplementary information): please specify the number of variants called in total and within each gene.

We have added this information to the last paragraph before “Statistical and Bioinformatic Analyses”.

- supplementary data 1: could you include the annotation of the selected smMIPs? i.e. the BOCA, and ENCODE characterization criteria that were used for selection.

Thank you for this suggestion – we have added a second tab to supplementary table S1 which lists the targeted genomic regions and their annotations as either exonic or identified by either BOCA or ENCODE.

- page 12, last paragraph. Please specify how many MIP sequence variants and samples passed QC.

We have added the number of variants passing QC in each of the 5 genes to the last paragraph before “Statistical and Bioinformatic Analyses”. The number of samples passing initial QC is already listed there, and the total number successfully genotyped for each variant is now listed in the Supplemental Tables S6 and S7.

- please also provide also references to specific software and state the used versions

Thank you for this reminder – we have added citations for the software used in the Statistical and Bioinformatics Analyses section.

- supplementary tables S6, S7: please also provide the allele count for the common variants.

Thank you for pointing out this omission. We have added two columns giving the total number of samples both phenotyped and genotyped, and the minor allele count, for each of the six phenotypes. This adds 12 columns to the table, but as it is an excel file, readers can manipulate it as they wish.

- results:
- it would be interesting to include annotations from JASPAR into supplementary tables 6 and 7. For instance, the main text specified that rs142310124
is the best candidate for the DCDC2-KIAA0319 haplotype, because it's predicted to disrupt motifs for four different TFs. However, this data is not available to the reader (which may want to evaluate other potential annotations as well).

Thank you for catching this omission. We have added a column of TFs whose binding sites are predicted to be disrupted, to Table 2, which is the list of significant findings. Supplementary Tables S6 and S7 list all common SNPs regardless of significance. We don’t feel there is any value added for the reader by listing TFs for SNPs that are not significantly associated with any of our phenotypes.

- Tables S6 and S7: please also specify what A1 and A2 are, and whether the frequency(A1) refers to the current sample of any reference population allele frequency.

Thank you for pointing out this omission – we have added the definitions of A1 and A2 to the top of Tables S6 and S7 and we added a Table S9 with the counts of individuals scored and frequencies obtained at each SNP. A1 and A2 are the rare and common alleles respectively, in this data set. As part of making Table S9, to clarify, we also added a mapping between the REF/ALT alleles, as are derived as part of the genotype calling, and the A1/A2 labels that we used for analysis and presentation. In putting the information into S9, we removed the frequency column from tables S6 and S7 because it changes (only very slightly) for each phenotype (due to tiny amounts of missing data). The reader can calculate the frequency from the MAC and n columns. We chose not to add frequency columns for each phenotype because it is redundant, and the tables are large.

- SNP rs55712458 is multiallelic (G/A/C). I think these other annotations should also be included somewhere.

This SNP is diallelic in our sample. The other alternate allele is vanishingly infrequent (1 in 67926, according to Gnomad v4.0.0), so we do not think there is value in mentioning it.

- it would be good to mention prior targeted sequencing efforts in the introduction, and to discuss the lack of replication with the KIAA0319 SNP rs138160539 from Caly et al. (2023). At the moment this lack of replication is only mentioned in the results section (page 18, lines 386-387).

We have added mention of this study to the introduction. The Cali paper did not assess statistical significance (due to the tiny sample size) so our failure to find an effect is not a failure to replicate. We have removed the sentence about the Cali exome variant from the results – the Cali study is better represented by the sentence we added to the introduction.

*A recent sequencing study (89) of specific learning disorders that included genes in DYX1 and DYX2 noted the existence of an exome variant in KIAA0319, but the small sample size (37 people) did not allow assessment of statistical significance.*

- page 22, last paragraph: the results referring to supplementary tables S4 and S5 refer to the SKAT-O analysis, but the methods sections mentioned both SKAT and SKAT-O (pages 14-15, lines 303-309). What are the results from the SKAT? if different to SKAT-O, does that inform about the potential assumptions (since the power of the tests is different, i.e. SKAT-O "is more powerful when most variants are causal and effects are in the same direction" (lines 307-308)?)

We did not use the SKAT test - we only used SKAT-O, which is the best approach to maximize power in this situation. We have added a phrase to the end of the second association testing paragraph (page 15) to clarify this:

*and is therefore the best choice in this situation where we do not have an a priori expectation of the direction or size of variant effects.*

- discussion:
- I think it would be informative to relate the current results to the recent larger-scale GWASes for dyslexia and reading-related quantitative traits (Doust et al., 2022 and Eising et al. 2022). What can we interpret from the current study and associations in common variants such as rs55712458 (MAF~0.2) that were not significant in those GWASes?

Both papers are referenced in the introduction.

In the Doust paper, the phenotype under study is self-reported dyslexia. In contrast, our phenotypes are measured with specific instruments administered by professionals and are thus more likely to be comparable to measures in used in other, similarly-evaluated, samples. The Doust paper did not find any signals in any of the previously identified dyslexia regions, including DYX1 and DYX2, which casts doubt on the utility of their definition of affected status.

The Eising GenLang paper uses similar phenotypes to ours in multiple large cohorts. However, their genotype data consists of GWAS SNPs, rather than sequence data. The SNP we identified, rs55712458, while common, does not appear on any of the Affymetrix or Illumina chips, so was not directly queried in the GWAS study. We have added a sentence to this effect to the discussion.

page 26, lines 513 onwards: the initial linkage report was with nonword repetition, while the current study finds an association with spelling but not nonword repetition (despite being partly the same sample?). Please provide a possible explanation for this.

We have elaborated on the differences between the two samples and the differences in statistical analyses.

*While the UW cohort here includes the samples that gave a signal on chromosome 12p for non-word memory, the statistical analyses of the two data sets are different. The original finding was based on linkage analysis, which is sensitive to rare alleles. In the analysis presented here, rare variants are analyzed in aggregate, and the precise choice of grouping for variants can affect the results. In addition, this analysis includes two other cohorts, which may weaken the association that led to the initial finding.*

Again, thank you for your help in improving this manuscript.

Sincerely yours,

Wendy H. Raskind, M.D., Ph.D.
