# Supplementary material for: Targeted analysis of dyslexia-associated regions on chromosomes 6, 12 and 15 in large multigenerational cohorts
Source: PLoS One. 2025 May 27;20(5):e0324006. doi: 10.1371/journal.pone.0324006 (PMC12112411; doi:10.1371/journal.pone.0324006)
Supplement: Attachment — Submitted filename: Chapman MIPseq Response to Reviewers 21Aug2024.docx [file pone.0324006.s008.docx]

**ITEMIZED RESPONSES TO THE CRITIQUES**

**Comment from Dr. van den Boer**

“The reviewer also suggests additional analyses. If it would be possible to run these and include them in the manuscript as suggested, I believe that would be very helpful. However, I understand that the authors might feel otherwise. Alternatively, authors should clarify the criteria used to define dyslexia across cohorts and discuss the potential implications thereof.”

We do not feel that additional analyses would overcome the limitations engendered by inclusion of cohorts from multiple study groups. Differences in ascertainment, inclusion criteria and phenotyping batteries are part and parcel of such collaborations. As such, covariate adjustments are a standard and expected approach to data analysis. We included such adjustments for cohort, with and without adjustment for VIQ, which should address differences in ascertainment by site as well as inclusion criteria for probands. We also did not carry out analyses of dyslexia per se, but instead analyzed the phenotypes as quantitative traits. Therefore, a description of details of dyslexia diagnosis across the cohorts does not have an obvious goal that is relevant to interpretation of the analyses. Details of ascertainment for the two larger studies are also available in cited papers, so any description in this paper would be duplicative. Analyzing only UW families in which the proband met a non-discrepancy criterion would also not be illuminating as the smaller sample size could simply lead to false negative results on the basis of sample size alone.

**Comments from the new reviewer**

1. “The study conducts a deep sequencing analysis for five candidate genes selected because of previous "strong support" (line 89) from the literature.”

We actually did not, and do not, state that the candidate loci had particularly strong support. We agree with the reviewer that this would be a misstatement of the evidence. The phrase “a small number have the strongest support’ was meant as a comparative. To address the reviewer's comment, since other readers may jump to the same conclusion, we changed that phrase to “a small number have received support by more than one group.” We also added a sentence in the last paragraph of the Introduction to clarify the choices of loci for analysis, “The analyses focused on two highly cited loci and a genomic region implicated by our previous studies and supported by the literature.”

“However, it's essential to acknowledge that previous associations at these loci may have derived from small and underpowered studies, which did not replicate in more recent GWAS studies.”

Failure to detect some variants of interest in any of the regions analyzed could also be explained by the limited sample size for carrying out association analyses.

“The authors recognise that there are inconsistent results in the literature but the manuscript should further emphasize the potential limitations of the original discovery studies and recognize the possibility that the associations in the selected genes could be false positives.”

We specifically cited failures to support the results of each type of study – linkage, association and GWAS. The concern about false positives holds for every linkage study, every GWAS, and every other statistical test, so it is not clear what the reviewer is asking for. Of these, when carried out properly, linkage analysis has a low false positive rate in sample sizes that are far less than those needed to carry out GWAS. But we undertook this comprehensive sequencing study precisely to study the question of “false positives”. As none of the previous sequencing studies of these genes/loci evaluated this large a sample nor included noncoding DNA the possibility of an undetected causative variant in any of the candidate genes could not be excluded. To be more explicit, we added a phrase and several sentences (highlighted below in yellow).

While support for involvement of the aforementioned genes has been reported from both a variety of association and linkage analyses and functional studies, evidence favoring particular genes in the ROIs is inconsistent or difficult to interpret [69-75]. There have been failures to detect linkage [76-78] or association [79-85], as well as reports of increased risk attributed to opposite alleles [50, 51, 79]. For a complex trait there is also the chance that composite quantitate trait loci (QTLs) are responsible for some of the linkage analysis results [86, 87]. False-positive results are another possible explanation. Demonstration of potential functional competence of the putative risk allele in an animal model is also difficult to interpret in the context of a human trait [88].

2. “The explanation offered at lines 108-112 to explain the different outcomes across studies is not convincing. First, it needs to be supported by references and second, while different criteria could have affected results outcome, the small sample sizes remains the most plausible explanation.”

We have reworded this portion of the paragraph and provided references.

“Variability in conclusions across the different study designs and samples is common and not surprising. Genetic heterogeneity has been responsible for discrepant results since the earliest days of genome scans, even for “simple” Mendelian traits [100]. Genome-wide linkage analyses and GWAS both allow location scans, but with different localization resolution and sensitivities to less vs. more-common trait-gene allele frequencies [101], and with power to detect genetic effects influenced by sample ascertainment procedures [102].”

Different statistical approaches require different sample sizes of participants, so many of the linkage studies of dyslexia or component phenotypes were equivalent or greater in power under some circumstances to some of the GWAS studies in this regard. The largest GWAS was flawed in using only self-report. To address the reviewer's concerns, we have included this statement: “However, the large sample size was only feasible though use of cases without a clinical diagnosis. This a situation that can lead to statistical heterogeneity in results, raising concerns about usefulness of such samples, as has been reported in application to another complex trait [98]. “

3. “Furthermore, the criteria for selecting these genes could be elucidated more clearly in the manuscript. While the study analyses 5 genes, the introduction describes previous associations only for DNAAF4, KIAA0319 and DCDC2. The rational for selecting GRIN2B and CYP19A1 is mentioned only later in the discussion. From what reported, the evidence for CYP19A1 is limited to one breakpoint study and GRIN2B was selected because of previous association in the UW cohort. As described by the authors, GRIN2B has been reported for associations with a range of cognitive traits including in GWAS, so it is possibly the gene out of the five selected is the one supported by the most strongest associations but these would not be specific to dyslexia. My suggestion is to reframe the study and explain the rational for selecting all five genes in the introduction. It is essential to be completely transparent around the weak evidence supporting these genes, that nonetheless featured prominently in the field of dyslexia genetics.”

In the Introduction, we added the rationale for selecting GRIN2B and CYP19A1 and cite the relevant references. “*CYP19A1,* another candidate gene in the DYX1 locus [37, 52] is of interest because of the almost universally observed ratio imbalance of males:females with dyslexia; CYP19A1 codes for aromatase, an enzyme that converts androgens to estrogens in the brain [53].”

The previous version had “From the other four ROIs identified by our SNP linkage analyses in the UW cohort, we chose to include the gene for ionotropic glutamate receptor subunit 2B (*GRIN2B*)”. We substituted: “Our linkage analyses for various quantitative measures of dyslexia using the University of Washington cohort identified additional candidate loci [63-65]. One of the strongest linkage signals was in a region on chromosome 12p [64], which contains *GRIN2B*, a gene that had support as a dyslexia candidate gene from studies in other data sets [66-68].”

4. “More explicitly, I would not start from the assumption that these genes are strong candidates but I would reframe the study as a comprehensive replication to further assess their potential role.”

We do not understand what is requested beyond what we had already written about our intent in the Introduction. We added the word “possible” in this sentence, “We carried out a comprehensive analysis of the coding region and some regulatory element motifs of five putative dyslexia risk genes to assess their possible role.” The word “potential” had already been used in a sentence in the same paragraph, “to investigate the potential role of variants.”

5. “Another issue is around the criteria for defining dyslexia. While it appears that the FLDRC and SickKids cohorts used the similar criteria based on cut-off on IQ and reading measures, the UW cohort applied a different criteria that consider IQ discrepancies. I do not agree with this criteria but I do understand that this is a topic open for debate. My suggestion would be to state clearly how many participants had scored below -1.5 SD from the mean on reading measures as in the two other cohorts.”

Please see our response to Dr. van den Boer.

In the Methods section we provided more explanation of how our strategy, including analyses of quantitative traits, minimized the problems inherent in joint analyses of independently collected study groups. “This strategy provided a large total number of participants screened with an essentially equivalent test-battery allowing for joint analysis across the three cohorts while minimizing introduction of excess phenotypic heterogeneity that may be introduced by mixing multiple phenotypic measures and including a threshold to create a binary affected/unaffected outcome. Even so, some variability in underlying risk allele frequencies is expected across cohorts here because details of recruitment invariably differ across recruitment sites, as is the case for virtually all analyses that aggregate data from multiple sites. This may lead to biased estimates of risk-allele effects (115) but does not affect interpretation of the results of hypothesis testing.”

6. “Ideally, the statistical analysis should be repeated with the exclusion of the participants that did not meet these criteria and presented in the supplementary material for the benefit of the reader that would have different views on the definition of dyslexia.”

Please see our comment to Dr. can den Boer.

7. “Finally, my main interpretation of the results is that the study does not robustly support the role of these genes in dyslexia, consistent with the interpretation that the initial discovery studies for these genes were false positives. While it is worth reporting the trends of observed associations, overall there is no compelling evidence. By addressing point 4) above and spelling out the weak associations in support of these genes, the main conclusion of the present study seems to be that these genes are unlikely to play a major role in dyslexia.”

We do not state that the genes described here either do or do not play a major role in dyslexia. Our study here is not appropriate for evaluating this question. We only report that there is strong statistical support for some role in the quantitative traits evaluated for some variants investigated here. Regardless, our study by itself does not negate the candidate locations as different genes might be responsible for the signals in these loci obtained in previous studies.

8. “The latest GWAS for dyslexia have demonstrated the highly polygenic nature of this condition. …. Therefore, rather than interpreting the results on the assumption that the selected genes are expected to play a major role with a specific class of genetic variants, it is necessary to recognise the highly polygenic nature of dyslexia (as opposed to the proposed “oligenic” model – line 60) and to contextualize the findings within our current understanding of the field of dyslexia, and more generally complex traits, genetics.”

Even if multiple genes participate in a phenotype, this does not preclude identification of one or more of them that contribute enough to be detected above the rest. GWAS and linkage analyses have different strengths and usages; the GWAS results do not negate the results of the linkage analyses and vice versa. Rather than using either oliogogenic or polygenic in the Abstract we have substituted “multigenic”.
