# Supplementary material for: Targeted analysis of dyslexia-associated regions on chromosomes 6, 12 and 15 in large multigenerational cohorts
Source: PLoS One. 2025 May 27;20(5):e0324006. doi: 10.1371/journal.pone.0324006 (PMC12112411; doi:10.1371/journal.pone.0324006)
Supplement: Attachment — Submitted filename: Chapman MIPseq Response to Reviewers 17 March 2025.docx [file pone.0324006.s009.docx]

**ITEMIZED RESPONSES TO THE CRITIQUES**

**Comment from Dr. van den Boer**

1. More clarity is needed about the inclusion criteria across the different samples. One of the samples seems to have applied a discrepancy criterion, whereas the other two focused on severity of the literacy impairments. This should be explicitly mentioned.

In the section in “**METHOD**S” on sample acquisition we have from the beginning specified the different inclusion criteria for probands. For additional clarity, in the first paragraph of the section “*Sample and phenotype selection strategy*” we added citations that provide detailed ascertainment and inclusion strategies for the University of Washington (UW) and The Hospital for Sick Children cohorts. In the individual site paragraphs, we refer to the “Supplement” where details from those publications can be found and mention the tests that were used in the qualification process. For example, the statement for the UW site reads: “Recruitment and evaluation …..are comprehensively described elsewhere, are briefly summarized herein, and are provided in more detail in the Supporting Information document.”

After that statement, as requested, we added this sentence: “For the UW cohort a discrepancy criterion was used for qualification of a child as a proband.”

2. The authors argue that this difference is covered by adding VIQ to the analyses, but that is incorrect. It is not IQ measures that are likely to differ, but literacy scores. Through a discrepancy criterion persons with less severe reading and/or spelling problems might have been included in one sample as compared to the others. The implications of these differences should be discussed in the manuscript.

There seems to be a confusion about both the rationale and analysis steps that we used and the points we were trying to make. In no case did we analyze IQ measures against any of the genomic variants. In all our association testing we look at correlation between variation in the covariate-adjusted literacy scores and the DNA variants/genotypes. To clarify what we did, we have re-organized and re-edited a number of paragraphs in the section “**Statistical and Bioinformatic Analyses**”**.** For readers who are not familiar with the standard statistical methods used in genetic epidemiology we added an overview that contains a brief description.

The section “*Phenotypes and adjustments*” has been extensively edited and rearranged to make it easier for those without substantial statistical expertise to understand when each component of the modeling is introduced.

Regarding the concern about use of a discrepancy score in probands’ ascertainment in only one site, this is only one example of an ascertainment differences among the three sites, as is typical of virtually all multisite projects. For this reason, we do our covariate adjustment within site to account for any site-specific effect. After all, the age-normalized score is an example of this to begin with - the actual reading scores that we start with are already re-scaled to reflect age- or grade-based expectations. However, there is ample reason and evidence in the literature to expect that VIQ measures may be correlated with the literacy scores, justifying investigation or comparison of the effect of association of VIQ-adjusted vs. not-adjusted *literacy scores* with genomic variants in the region. The value or necessity of including (or not) IQ or VIQ has different "camps" in the dyslexia research community, and we are not married to either camp. We feel that especially as a PLoS One submission it is best to provide both analyses, although our preference is to lean more heavily on results from the UNADJ analyses with their larger sample sizes.

To the extent that VIQ and any of the traits are correlated, adding the VIQ of individuals to the analysis model does adjust for this sampling detail because in the end the analysis investigates association of the residuals from the linear mixed model (where the VIQ for an individual is a fixed effect covariate) and number of ALT alleles at each SNV. The VIQ is never evaluated directly in an association analysis to the SNV ALT allele count, as the critique seemed to imply.

A fact that is very important for interpretation of our results is that we did not assess association of the variants/genes to dyslexia but only to quantitative phenotypes (endophenotypes) used to assess reading ability/impairment. We think we have diminished the possible confusion by re-editing the analysis section and inserting clarifying phrases throughout. The first sentence of the “**DISCUSSION**” already read “Here we provide results of a comprehensive investigation of underlying genomic variation in and surrounding five genes with prior evidence for an inherited effect on endophenotypes of dyslexia risk.” We have added phrases in several places to make this more apparent throughout. The “**ABSTRACT**” now substitutes “traits” for the quantitative scores on measures used to assess dyslexia, “We did not analyze dyslexia *per se*”. In the “**INTRODUCTION**” we added a phrase to the sentence beginning “Here we carried out a comprehensive analysis … to assess their possible role”. It now ends with “in performance on six tasks commonly used in the evaluation for dyslexia.” In “**METHODS**”, under “*Sample and phenotype selection strategy*” we state, “We only used the quantitative phenotypes and not the dyslexia diagnostic status for our analyses; henceforth, we use “trait(s)” to refer to these quantitative measures.” To the first sentence under “**Statistical and Bioinformatic Analysis**” we added: “We carried out a comprehensive association analysis of performance on six tasks commonly used in the evaluation for dyslexia, but did not analyze dyslexia *per se*.” For the remainder of the manuscript and supplement, we substituted “trait” for “phenotype” in all relevant places to refer to adjusted quantitative phenotypes.

In the “**DISCUSSION**”, to the sentence beginning “Targeted sequencing of two genes in the dyslexia-risk locus *DYX1* on chromosome 15 provides no support for a role for *DNAAF4*” we added the phrase “in modulating performance on any tested trait”. And n the last sentence of the paragraph on *CYP19A1*, after “A possible role for this gene in dyslexia” we inserted the phrase “and reading/spelling performance on traits related to dyslexia”.

In “**METHODS**” at the beginning of the section “*Phenotypes and adjustments*”, we added an explanation for why we included participant sets ascertained with different criteria. “As with all complex traits for which there is some heterogeneity across collection sites, the resulting potential heterogeneity adds a cost to the sample size required to detect association by reducing variant effect size in the full sample. However, the only way to achieve sufficiently large samples to detect association with complex traits is to include as many existing sample sets as possible that have that have assessed the same traits.”

3. In addition, I would appreciate more information in general on the ranges of scores obtained in the included participants. Authors argue that an important limitation of previous studies is that participants were included without a formal diagnosis of dyslexia. However, this also seems to be the case in the current study. As families were included, not every member of the family would have dyslexia or risk scores on literacy right? I realize that this last question is probably related to me being rather unfamiliar with this type or research. However, as this is probably the case for more readers, I would still appreciate some more information on this in the manuscript, without readers having to resort to previous publications.

You are absolutely correct that not all family members have dyslexia, but all family members who were used in the analysis do have phenotype data for at least some of the traits in “Table 1”, and these data are used. It seems as if this nuance might have been unclear, despite the numbers and symbols in “Table 1”. We have added an additional column to “Table 1” to summarize the families a bit more and inserted more explicit statements to the “**METHODS**” under the “**Statistical and Bioinformatic Analysis**” section. As requested, we also added a new summary table to the supplement (Table S4) with more summary statistics for each of the "raw" (age-normed) variables.

In terms of subject sampling, choice of immediate (first-degree) family members for measurement of phenotypes and from whom to obtain samples for DNA isolation was purely based on those subjects' availability. It is not at all unusual in genetic studies that complete sibships and both parents are sampled and measured when the trait under study is identified through children. But our intent was not to write a review paper about how to carry out a genetic study, which is what it would take to cover every detail of how one does such studies. There is plenty of existing literature (and books) to help the novice get started.

Regarding the range of scores in all three cohorts it is important to consider that ascertainment was done through a proband, but parents and siblings were included regardless of history of dyslexia/reading difficulty or performance on the tests. The probands constitute the minority of subjects in the analyses.

To make it clear from the outset that we evaluated association of the genes with phenotypes used to assess dyslexia, not dyslexia itself, beginning with the “**ABSTRACT**”, we use the term “traits” for the quantitate phenotypes throughout.

In addition, in the last paragraph of the “**INTRODUCTION**” we reworded the sentence that previously read: “Here we carried out a comprehensive analysis of the coding region and some regulatory element motifs of five putative dyslexia risk genes to assess their possible role.” It now reads: “We report results from a comprehensive analysis of the coding regions and some regulatory element motifs of five putative dyslexia risk genes to assess their possible role in performance on six tasks that yield quantitative scores and are commonly used in the evaluation for dyslexia.” This change should clarify that we assessed dyslexia-related phenotypes, not dyslexia itself.

4. I think it is now sufficiently clear in the introduction that previous evidence on dyslexia-related genes is weak at best and that the current study is focused on some that have previously been associated with dyslexia. However, what is missing is a theoretical or more fundamental understanding of why particularly these ROIs would be of interest. This has been added for some of the ROIs, but not all. To me, it now comes across as a rather random selection of RIOs, whereas this is probably not the case. See also points 1 and 2 of the reviewer.

**Comments from Reviewer 3**

1 (a). The criteria for gene selection is still unclear and unconvincing. Although, I appreciate that the authors now specify at the end of the introduction that the genes were selected as “highly cited loci” the abstract also states that the genes were selected on the bases of prior evidence of “association from more than one samples”.

In the sentence excerpted above, our use of the word “association” was confusing because it can be used to imply “involved in some way” but has a specific meaning as a statistical approach. All candidate genes included in our study were identified/nominated through further research in regions initially identified through linkage analyses. To avoid this imprecise wording, we replaced the “of association” with “for a role”

1 (b). Such criteria and such evidence have not been fully clarified. Specifically, there seem to be confusion between broad loci identified through linkage analysis and genes proposed via candidate gene association studies. For example, multiple studies reported linkage at DYX1, but only one study reported association for the CY19A1 gene.

The “**INTRODUCTION**” lists the types of analyses that led to identification of the loci (linkage analyses, genome-wide association studies, copy number scan, structural chromosome rearrangements and whole genome sequencing). We now provide more information regarding the choice of the genes to target within the loci. This is discussed in our response to point 2 below.

2) My impression is that the five genes were mainly selected because reported in the literature, but no specific criteria were applied.
If that is the case, this needs to be spelt out more clearly.

This was a first foray into examining a relatively complete set of variants at the sequence level data in a large sample of learning disabilities data sets. We chose a strategy that balanced cost against the impossible task of a complete genome wide scan, which would have been too much to try for a complex trait at this point with this sample size. We realize that the organization of the manuscript made it difficult to grasp the reason for selection of the 12p locus containing the *GRIN2B* gene. In the “**INTRODUCTION**” we provide some more information about the loci and in the “**METHODS**” section “**Overview of Rationale and Data Used**” we added two paragraphs to explain the logic behind the number and choice of candidate genes. In the first paragraph contains this wording:

“Practical issues of number of genes investigated were driven by cost, sample size, and challenges of interpreting genomic sequence data in non-coding sequence. To maximize sample size, we sequenced every individual in our combined dataset who had the relevant phenotypic data. This strategy gave us capacity to evaluate five genes and regulatory/splice regions around those genes in three genomic regions.”

The second through fourth paragraphs give details of the choice of regions and genes and has additional literature citations.

3 (a). My view is that the evidence supporting these genes is very weak and the revised manuscript and the response of the authors have not changed my position.
In particular, when highlighting that these genes are not supported by GWAS results, the authors suggest in their response that the selected genes have been identified via linkage studies for which large samples are not necessary. This is problematic because, while most of these genes were selected as candidates for being located within linked regions, they were tested mainly through association analysis. It is now well established that association studies in small samples are likely to lead to false positive results.

We make two comments here. First, virtually *all* gene identification involves association analysis at some stage. This is only one of the possible steps and processes used to get from a genome region to a gene. There are the rare exceptions where a mutation falls in an obvious gene, but because we don’t know a lot about what most genes do, this is an uncommon situation. Second, the comment about small samples and false positive results is not complete. Yes, one can get false positive results, but also one can get false negative results. One might simply not recognize the false negative results because of how one does literature searches, and false positive results are more likely to make it to publication. But the statement as it stands is incorrect at the core of statistical analysis.

3 (b). Furthermore, in their revision the author argues that the lack of clinical diagnosis could have affected the large GWAS by Doust et al. However, earlier GWAS that used both clinical diagnosis (Gialluisi et al 2021 [https://www.nature.com/articles/s41380-020-00898-x](https://urldefense.com/v3/__https:/www.nature.com/articles/s41380-020-00898-x__;!!K-Hz7m0Vt54!nnzZre2lFCuqwB1PTKx-cu-eGYRuOOBkjGmYRq7sNDCSd7hVj7kEQ9q8qH7g_W7xADABXeT6HUUgCgF-$)) and quantitative measures (Gialluisi et al 2019) also failed to provide support for the genes selected here. Notably these GWAS analysed samples that led to the identification of some of the dyslexia linked regions.

Our selection of the regions and genes preceded the publication of the paper by Doust *et al*, 2022 and both papers by Gialluisi *et al*, and results from these papers do not change our strategy. Sequencing of samples was also in progress before any of these papers were published. We note, also, that 4 of the 5 genes that we looked at were among the subset of 8 genes that Gialluisi *et al* 2019 evaluated (DYX1C1, KIAA0319, DCDC2, GRIN2B and DNAAF1=DYX1C1), with similar reasons for gene selection to ours, and with both groups making this decision independently. So, it is hard to reconcile that observation with the comments by this reviewer, unless the reviewer is confused about what results from a GWAS can and cannot tell us.

There is a large literature in genetic epidemiology and statistical genetics and even excerpting a portion of it would not be appropriate for this research paper. However, to underscore our comments above, we provide in this response concrete examples of failure of GWAS to detect genes of interest from the research community of Alzheimer’s disease (AD, which one of us has worked in for ~38 years). As background, note that four well-established genes were identified in the early 1990s from linkage analysis followed by sequencing in the regions and targeted association analysis. This occurred long before the GWAS design was possible. Three of the genes have rare variants at multiple sites, and one gene (APOE) has common variants at two close SNPs, which creates the three alleles at this gene. APOE has a very strong association with AD: detectable in direct sequencing and single-gene testing in a few dozen subjects. The other three genes have multiple rare, very-high risk variants/mutations in them. Two observations are instructive. First, early GWAS failed to give positive results for anything near APOE. Given this failure around an important, known, gene, two key APOE SNPs were added to many SNP arrays. However, it turned out that only one of those SNPs returns results, and those results are still not very reliable, so knowledgeable groups do APOE genotyping or sequencing separate from GWAS arrays. Second, to date GWAS have not detected the other three genes, despite having very large samples. So again – GWAS works badly when a collection of rare variants affects a trait. Our investigation has tried to circumvent this latter issue by using an approach that works well for analyzing aggregate variants

Therefore, the revised interpretation for the lack in GWAS of support for the selected genes is not convincing and needs to be revisited.

We do not understand what the concern is. GWAS does not exclude presence of rare causal variants or variants that are not well tagged by the SNPs in the GWAS panel. We have not carried out a GWAS. We preformed targeted association analyses but evaluated much more of the DNA around a small number of heavily cited genes and gene regions. GWAS does not comprehensively investigate the DNA and does not work well with rare variation. We feel that we can put further consideration of one of the genes to rest – there is no evidence for association to any interpretable parts of the DNA in and around the gene. We could not conclude this from a GWAS because of its blind spots. We have provided all the results for all the positions sequenced so other investigators can meta-analyze our results with theirs if they choose. In this sort of investigation one study does not tell the final story, and it is important to be able to have multiple independent projects that look at the same question. PLOS ONE also states that statistical significance is not a requirement: ”*We evaluate research on the basis of scientific validity, strong methodology, and high ethical standards—not perceived significance.*”

4) In line with the weak evidence supporting these genes, my interpretation of the results is that, as expected, the present study does not support their role in dyslexia.

We think this interpretation unnecessarily simplifies what we have done and found. The coding portions of the genes in question were not comprehensively studied in many individuals previously and the potentially regulatory regions near the genes were mostly ignored, leaving open the possibility that causative/contributory variants might have gone unrecognized. By carrying out the targeted sequencing, we have been able to provide evidence *against* *DNAAF4* for the traits we analyzed. However, in and around some of the other genes we have positive signals that survive multiple testing corrections. These results need replication, which we hope publication of our study will stimulate.

Other points:

- Please, avoid referring to dyslexia as a “disability” or “disorder” and use terms like “difficulty”.

Various wordings are used to define “dyslexia”. It is commonly described as a learning disorder or learning impairment. DSM 5 includes dyslexia as a specific learning disability affecting reading — not just a learning difference reading — and there is an ICD-11 code, 6A03.0, that refers to "Developmental learning disorder with impairment in reading". In the field of Medicine, the use of the word “disorder” or “disability” is not pejorative. These terms make it more likely that a person will get resources to help with the problem. We have substituted the word “impairment” for “disability”.

- The submission states that “All data are fully available without restriction”, but I was not sure where to find them.

We provide results of all the analyses (at the variant level) in tables in the Supplement. If all the variant genotypes or the raw sequence data (the vcf files) are requested, we will submit these files to dbGaP.
